# Determining the status of tertiary lymphoid structures in invasive pulmonary adenocarcinoma based on chest CT radiomic features

**DOI:** 10.1186/s13244-025-01906-w

**Published:** 2025-01-29

**Authors:** Ye Yu, Tianshu Yang, Pengfei Ma, Yan Zeng, Yongming Dai, Yicheng Fu, Aie Liu, Ying Zhang, Guanglei Zhuang, Yan Zhou, Huawei Wu

**Affiliations:** 1 Department of Radiology, Renji Hospital, School of Medicine, Shanghai Jiao Tong University, Shanghai, China; 2 State Key Laboratory of Oncogenes and Related Genes, Renji-Med X Clinical Stem Cell Research Center, Ren Ji Hospital, School of Medicine, Shanghai Jiao Tong University, Shanghai, China; 3 Department of Research Center, Shanghai United Imaging Intelligence Co., Ltd., Shanghai, China; 4 School of Biomedical Engineering, Shanghai Tech University, Shanghai, China; 5 Shanghai Key Laboratory of Gynecologic Oncology, Ren Ji Hospital, Shanghai Jiao Tong University School of Medicine, Shanghai, China

**Keywords:** Radiomics, Tertiary lymphoid structures, Adenocarcinoma of lung

## Abstract

**Objectives:**

The aim of this study was to determine the status of tertiary lymphoid structures (TLSs) using radiomic features in patients with invasive pulmonary adenocarcinoma (IA).

**Methods:**

In this retrospective study, patients with IA from November 2015 to March 2024 were recruited from two independent centers (center 1, training and internal test data set; center 2, external test data set). TLS was divided into two groups according to hematoxylin-eosin staining. Radiomic features were extracted, and support vector machine (SVM) were implemented to predict the status of TLSs. Receiver operating characteristic (ROC) curves were used to analyze diagnostic performance. Furthermore, visual assessments of the test set were also conducted by two thoracic radiologists and compared with the radiomics results.

**Results:**

A total of 456 patients were included (training data set, *n* = 278; internal test data set, *n* = 115; external test data set, *n* = 63). The area under the curve (AUC) of the radiomics model on the validation set, the internal test set, and the external test set were 0.781 (95% confidence interval (CI): 0.659–0.905;), 0.804 (95% CI: 0.723–0.884;) and 0.747 (95% CI: 0.621–0.874;), respectively. In the visual assessments, the mean CT value and air bronchogram were important indicators of TLS, the AUC was 0.683. In the external test set, the AUC of the clinical model was 0.632.

**Conclusions:**

The radiomics model has a higher AUC than the clinical model and effectively discriminates TLSs in patients with IA.

**Critical relevance statement:**

This study demonstrates that the radiomics-based model can differentiate TLSs in patients with IA. As a non-invasive biomarker, it enhances our understanding of tumor prognosis and management.

**Key Points:**

- TLSs are closely related to favorable clinical outcomes in non-small cell lung cancer.
- Radiomics from Chest CT predicted TLSs in patients with IA.
- This study supports individualized clinical decision-making for patients with IA.

**Graphical Abstract:**

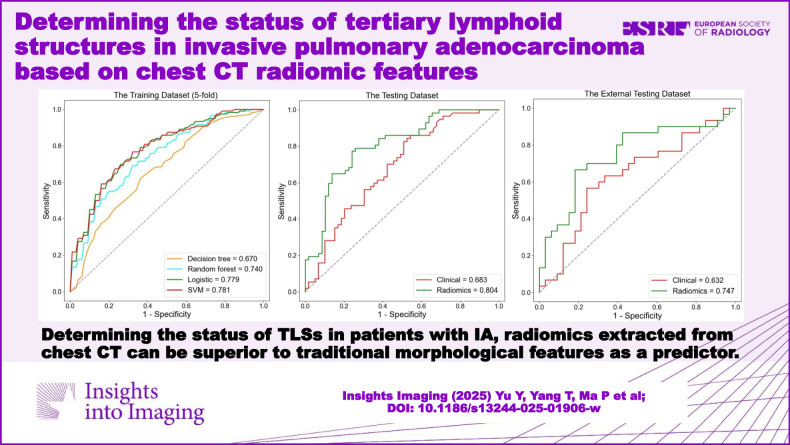

## Introduction

Lung cancer, globally acknowledged as a dominant malignancy, has an alarming incidence and mortality rate. Lung adenocarcinoma, in particular, represents the most prevalent pathological subtype [1]. According to the pathological classification standard of the World Health Organization, lung adenocarcinoma can be categorized into four types, including invasive pulmonary adenocarcinoma (IA) [2]. IA specifically, is characterized by rapid growth and a pronounced risk of potential metastasis, reinforcing the critical need for early intervention. Accurately predicting patient outcomes and prognosis after treatment for this type of lung cancer aids clinicians in making informed decisions during the diagnostic and treatment process, developing long-term treatment plans, and ultimately improving patient prognosis.

In recent years, tertiary lymphoid structures (TLSs) have been identified to exhibit a significant correlation with prognosis in non-small cell lung cancer, and have been evidenced to bolster anti-tumor responses and predict the efficacy of immunotherapy [3–7]. These ectopic lymphoid formations emerge in response to inflammation, infection, and tumor presence, characterized by organized colonies of T and B lymphocytes [8, 9]. The infiltration of TLS within the immune microenvironment of the tumor contributes to a favorable prognosis. Generally, cases with a higher TLS density, better infiltration degree, and greater maturity level have better prognostic outcomes. Hence, the assessment of TLSs has gained increasing importance due to emerging evidence supporting their prognostic and potentially predictive significance across various tumor types [7, 10–14]. However, an effective predictive method for TLSs remains elusive due to their noteworthy heterogeneity.

Noninvasive prediction of TLSs in IA has been greatly enhanced by the use of CT. CT imaging, rich in lesion information, enables non-invasive assessment and prediction of lesion types, treatment responses, and more, significantly streamlining the diagnosis and treatment process of IA. However, it primarily relies on the diagnostic experience of clinicians and demands substantial human resources. In contrast, radiomics is an innovative imaging tool that extracts high-throughput and high-dimensional features from CT images [15]. This technique constructs clinically focused imaging biomarkers to improve diagnostic or prognostic accuracy (ACC), aid in lesion characterization, and optimize patient surveillance strategies [16].

Evaluating TLSs requires a substantial amount of tumor tissue, which presents a challenge due to the current lack of efficient evaluation methods. In contrast, radiomics offers a more advanced approach, enabling detailed exploration of tumor tissue in vitro and providing potential advantages in managing IA. In this study, we aim to assess the status of TLSs using radiomic features in patients with IA.

## Methods

### Dataset introduction

**Fig. 1:**
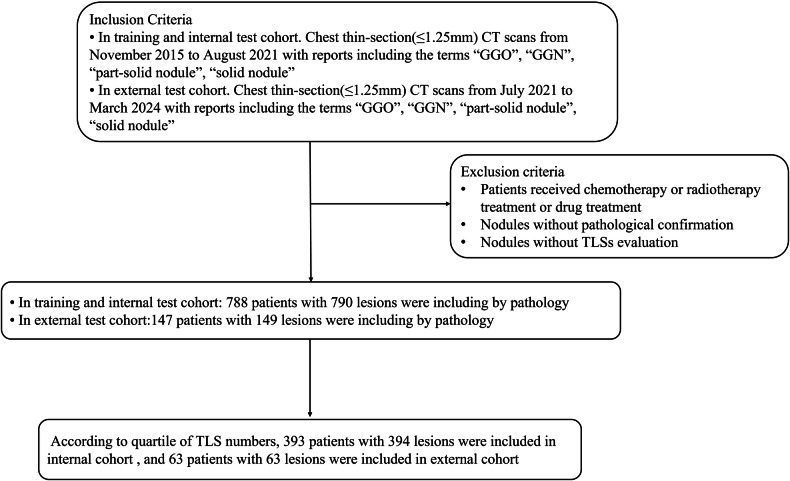
The flowchart of study patients. GGO, ground-glass opacity; GGN, ground-glass nodule

This retrospective study was approved by the Institutional Review Board and the requirement for written informed consent was waived. From November 2015 to August 2021, chest thin-section (≤ 1.25 mm) CT scans with reports including the terms “ground-glass opacity (GGO),” “Ground-glass nodule” (GGN), “part-solid nodule,” “solid nodule (SN),” or “subsolid nodule” were retrospectively reviewed. Finally, in total, 393 patients (145 men, 248 women, 62.40 ± 10.53 years) with 394 lesions were considered. From July 2021 to March 2024, 63 patients with 63 lesions were included in the external cohort. The exclusion criteria were as follows: (1) patients received chemotherapy or radiotherapy treatment or drug treatment; (2) nodules without pathological confirmation; and (3) nodules without TLS evaluation, as shown in Fig. 1.

Preoperative chest CT was performed using the Discovery CT750 HD, Discovery CT, LightSpeed VCT (General Electric Medical Systems), uCT760, uCT510 (United Imaging Medical Systems), IQon Spectral CT (Philips Medical Systems), etc. In the external set, the CT machine was Somatom Force CT (Siemens Medical Systems). The reconstruction slice thicknesses were 1.0 mm or 1.25 mm, and the intervals were 0.8 mm using a standard reconstruction algorithm. Unenhanced CT images of the latest CT examination before surgery were used. When contrast medium injection was required, only the pre-contrast image set was used. The mean interval between the latest CT examination and surgery was approximately 2 weeks.

### Histopathological indicators

For each case in this cohort, slides were assessed by two independent and experienced pathologists. The existence of intra-tumoral TLSs was evaluated morphologically on hematoxylin-eosin stained slides, which were scanned into whole slide images by using the ScanScope CS2 (Leica). According to a previous study, TLSs were classified into three groups, including aggregates of lymphocytes and lymphoid follicles with or without germinal center formation [17]. For this cohort, we classified tumors without any TLSs as the TLS-L (TLS-low) group, and tumors with at least ten TLSs as the TLS-H (TLS-high) group according to the upper quartile of TLS numbers.

### Image preprocessing and lung nodule segmentation

**Fig. 2:**
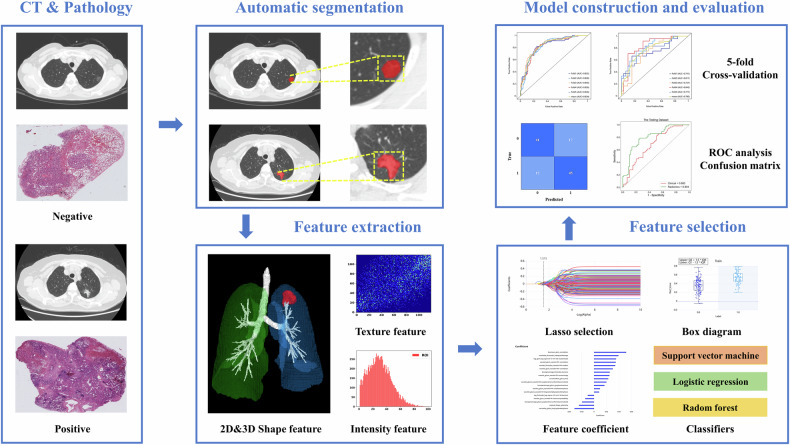
The radiomics workflow in the prediction of TLSs. CT, computed tomography*;* ROC, receiver operating characteristic

The workflow of radiomics consisted of image segmentation, feature extraction, feature selection, model construction, and evaluation (Fig. 2). We randomly divided 393 patients into training (*n* = 278) and testing (*n* = 115) at a ratio of 7:3, with a consistent TLS-H and TLS-L sample distribution. In the image preprocessing process, we first normalize the CT images by the *Z*-score normalization method, with the normalized intensity values truncated to the range [−1, 1] [18].

A deep learning segmentation model was built with VB-Net to automatically segment lesions. VB-Net is a modified 3D convolutional neural network that combines V-Net with bottleneck structures and is much faster than V-Net. In our previous research (segmentation results of VB-Net for the lung of 307 testing CT images, and nodules of 500 testing CT image patches) [19], the average dice coefficient of the model was (98.9 ± 0.4)% on the left and right lungs, and (91.5 ± 6.3)% on nodules. Before feeding the images into the deep learning network, we resampled each of the CT images to a spacing of 0.2 × 0.2 × 1.0 mm^3^. To avoid overfitting and increase the robustness of the deep learning network, image augmentation, including rotation, scaling, and flipping, was performed on each image with a probability of 0.5. Rotation was randomly performed with an angle along an axis in a range of −5° to 5°. The scaling factor was randomly sampled in a range from 0.75 to 1.25.

Two radiologists independently examined the region of interest of the segmented model, preserved the nodules with pathological and TLS labels, and performed a three-dimensional evaluation of nodules using ITK-SNAP (version 3.8.0, http://www.itksnap.org).

### Feature extraction

Radiomic analysis was performed with the uAI Research Portal (United Imaging Intelligence), which was embedded into the widely used package PyRadiomics (https://pyradiomics.readthedocs.io/en/latest/index.html). Radiomic features were extracted from the different regions (gross tumor volume, solid, ground-glass, and perinodular). Based on the original CT images, the following feature categories were extracted: (a) first-order statistics; (b) 2D and 3D shape features; and (c) texture features. Meanwhile, 15 imaging filters (e.g., LoG, Wavelets, Gaussian) were also used for feature extraction. Ultimately, 2264 features were extracted from each image.

### Feature selection and model construction

In all, 2264 radiomic features were extracted, and then the features were selected by the least absolute shrinkage and selection operator regression analysis algorithm. The performance reached the optimum when the tuning parameter Lambda (λ) was set to 0.03054 [20].

**Fig. 3:**
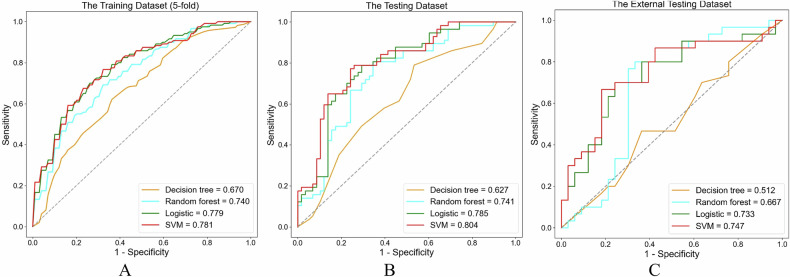
Receiver operating characteristic (ROC) curves of the training (**A**) and testing (**B**, **C**) (internal and external) set between different algorithms. SVM, support vector machine

In order to avoid the instability of the model performance due to the TLS-H and TLS-L sample deviation of the grouping, we adopted a 5-fold cross-validation method for the study population. More specifically, the whole population was randomly but equally divided into five groups, with the same proportion of TLS-H and TLS-L in each group, one group was selected as the test set for each experiment, and the other four groups were used as the training set for five times. For maximizing the discrimination of radiomics algorithm, the machine-learning classifiers of logistic regression, decision tree, random forest, and support vector machine (SVM) were implemented to model construction, respectively. In this study, SVM showed the best performance in terms of discrimination and was finally selected (Table 1). The ROC curves of the training and testing (internal and external) set between different algorithms are shown in Fig. 3.

**Table 1:** The classification results of ablation analyses on training, internal, and external test cohort

Methods	The training cohort				The internal test cohort				The external test cohort			
	AUC (95% CI)	ACC	SEN	SPE	AUC (95% CI)	ACC	SEN	SPE	AUC (95% CI)	ACC	SEN	SPE
Decision tree	0.670 (0.526–0.814)	0.619	0.517	0.697	0.627 (0.525–0.730)	0.600	0.491	0.707	0.512 (0.368–0.656)	0.508	0.300	0.697
Random forest	0.740 (0.609–0.873)	0.676	0.633	0.709	0.741 (0.650–0.833)	0.687	0.614	0.759	0.667 (0.525–0.809)	0.651	0.600	0.697
Logistic regression	0.779 (0.658–0.903)	0.723	0.658	0.772	0.785 (0.700–0.870)	0.739	0.737	0.741	0.733 (0.605–0.862)	0.667	0.533	0.788
SVM (final)	0.781 (0.659–0.905)	0.734	0.692	0.766	0.804 (0.723–0.884)	0.748	0.789	0.707	0.747 (0.621–0.874)	0.746	0.667	0.818

*AUC* area under the curve, *ACC* accuracy, *SEN* sensitivity, *SPE* specificity, *SVM* support vector machine

### Visual assessment

Visual assessments of the test set were also conducted by two thoracic radiologists. They were unaware of patient information and pathologic results and interpreted the images independently of each other, and their disagreement on assessment was resolved by consensus. Lobulation, spiculation, air bronchogram, pleural indentation, vascular convergence, vacuole sign, vascular going through, and interfaces of the lesions were assessed. The mean CT value and maximum nodule diameter were measured as well.

### Statistical analysis

All statistical analyses were performed with SPSS software (version 25, IBM Corporation) and R software (version 4.1.0, https://www.r-project.org). *p* < 0.05 was considered statistically significant, and all statistical tests were two-sided. To quantitatively evaluate the classification results, metrics including sensitivity (SEN), specificity (SPE), ACC, F1-score, weighted average F1 score (F1AVG = 2 × precision × recall/(precision + recall)), which balances precision and recall), Pearson correlation analysis was used between significant radiomics features and the TLS-H group and the receiver operating characteristic (ROC) curve was used to evaluate the performance of the model in differentiating TLSs between the training set and the test set. Shapiro–Wilk test was used to test normality. The independent sample *t*-test (conforming to the normal distribution) was used for the comparison of age, maximum nodule diameter and mean CT value. The Chi-square test was used to compare the counting data. The intraclass correlation coefficient (ICC) was used to assess the variates between the two radiologists in the visual assessment: generally poor: ICC < 0.2; fair: 0.2 < ICC ≤ 0.4; moderate: 0.4 < ICC ≤ 0.6; good: 0.6 < ICC ≤ 0.8; and excellent: 0.8 < ICC ≤ 1.0. Multivariate Logistic regression and backward step-wise selection were used to build the clinical model. DeLong’s test was used to compare the area under the curve (AUC) values of different models.

## Results

### Patient demographics and clinical characteristics in all cohorts

The flowchart of study patients is shown in Fig. 1. The clinical pathologic characteristics of all patients are summarized in Table 2. There were significant differences in location and tumor stages between the two groups (*p* = 0.004, *p* < 0.001).

**Table 2:** General clinical characteristics of 393 patients with 394 lesions

Variable	TLS-H group (%)	TLS-L group (%)	*p*-value
Sex, *n* (%)			0.474
Male	69 (38.98)	76 (35.18)	
Female	108 (61.02)	140 (64.82)	
Age, years (mean ± SD)	62.06 ± 10.37	62.68 ± 10.67	0.551
Location			0.004^*^
RUL	62 (35.03)	54 (24.89)	
RML	14 (7.91)	10 (4.61)	
RLL	32 (18.08)	34 (15.67)	
LUL	55 (31.07)	76 (35.02)	
LLL	14 (7.91)	43 (19.81)	
Nodule type			0.322
GGN	132 (74.58)	171 (78.80)	
SN	45 (25.42)	46 (21.20)	
Tumor stages			0.550
I A1–I A3	147 (83.05)	185 (85.25)	
I B–IVA	30 (16.95)	32 (14.75)	

*TLS-H* tertiary lymphoid structures high, *TLS-L* tertiary lymphoid structures low, *SD* standard deviation, *RUL* right upper lobe*, RML* right middle lobe*, RLL* right lower lobe*, LUL* left upper lobe*, LLL* left lower lobe*, GGN* ground-glass nodule*, SN* solid nodule

*p* < 0.05 is indicated in superscript asterisks for significance

### Demographic data, CT characteristics in the test cohorts

The demographic data, mean CT value, and morphological features in the test cohorts are listed in Table 3. The two radiologists achieved good to excellent agreement in the visual assessment (Supplementary Table S1). There was no difference in sex, age, or most morphological features between the two groups. There were significant differences in the maximum nodule diameter, mean CT value, and air bronchogram between the two groups (*p* = 0.015, *p* = 0.003, and *p* = 0.009). The cut-off mean CT value was −272 Hounsfield units (HU). The clinical model showed that the mean CT value (odds ratio (OR): 1.002; 95% confidence interval (CI): 1.000, 1.004) and air bronchogram (OR: 0.441; 95% CI: 0.203–0.960) were independent indicators of TLS (Table 4).

**Table 3:** Demographic data, mean CT value, and morphological features in the test cohorts

Characteristic	TLS-H group (%)	TLS-L group (%)	*p*-value
Sex, *n* (%)			0.955
Male	20 (35.09)	20 (34.48)	
Female	37 (64.91)	38 (65.52)	
Age, years (mean ± SD)	61.07 ± 10.58	63.19 ± 11.14	0.296
Location			0.391
RUL	25 (43.86)	16 (27.12)	
RML	2 (3.50)	2 (3.39)	
RLL	11 (19.30)	12 (20.34)	
LUL	13 (22.81)	19 (32.20)	
LLL	6 (10.53)	10 (16.95)	
Maximum nodule diameter (mm)	23.74 ± 10.58	19.05 ± 9.93	0.015^*^
Mean CT value (mean ± SD)	−169.40 ± 183.34	−285.99 ± 228.34	0.003^*^
Spiculation			0.428
No	21 (36.84)	26 (44.07)	
Yes	36 (63.16)	33 (55.93)	
Lobulation			0.357
No	7 (12.28)	4 (6.78)	
Yes	50 (87.72)	55 (93.22)	
Air bronchogram			0.009^*^
No	23 (40.35)	38 (64.41)	
Yes	34 (59.65)	21 (35.59)	
Pleural indentation			0.949
No	19 (33.33)	20 (33.90)	
Yes	38 (66.67)	39 (66.10)	
Vascular convergence			0.853
No	28 (49.12)	30 (50.85)	
Yes	29 (50.88)	29 (49.15)	
Vacuole sign			0.445
No	45 (78.95)	43 (72.88)	
Yes	12 (21.05)	16 (27.12)	
Vascular going through			0.186
No	13 (22.81)	20 (33.90)	
Yes	44 (77.19)	39 (66.10)	
Interface			0.472
Ill-Defined	24 (42.11)	21 (35.60)	
Well-defined	33 (57.89)	38 (64.40)	

*SD* standard deviation*, RUL* right upper lobe*, RML* right middle lobe*, RLL* right lower lobe*, LUL* left upper lobe*, LLL* left lower lobe

*p* < 0.05 is indicated in superscript asterisks for significance

**Table 4:** Diagnostic efficacy of parameters in determining the TLSs in IAC

Parameters	AUC	Threshold	SPE	SEN	*p*-value
Maximum nodule diameter (mm)	0.656	14.6	0.441	0.877	0.002^*^
Mean CT value (mean ± SD)	0.638	−272	0.593	0.719	0.008^*^
Air bronchogram	0.620	/	0.644	0.597	0.021^*^
Joint parameter	0.683	/	0.607	0.593	< 0.001^*^

*SD* standard deviation, *AUC* area under the curve

*p* < 0.05 is indicated in superscript asterisks for significance

### Feature selection

**Fig. 4:**
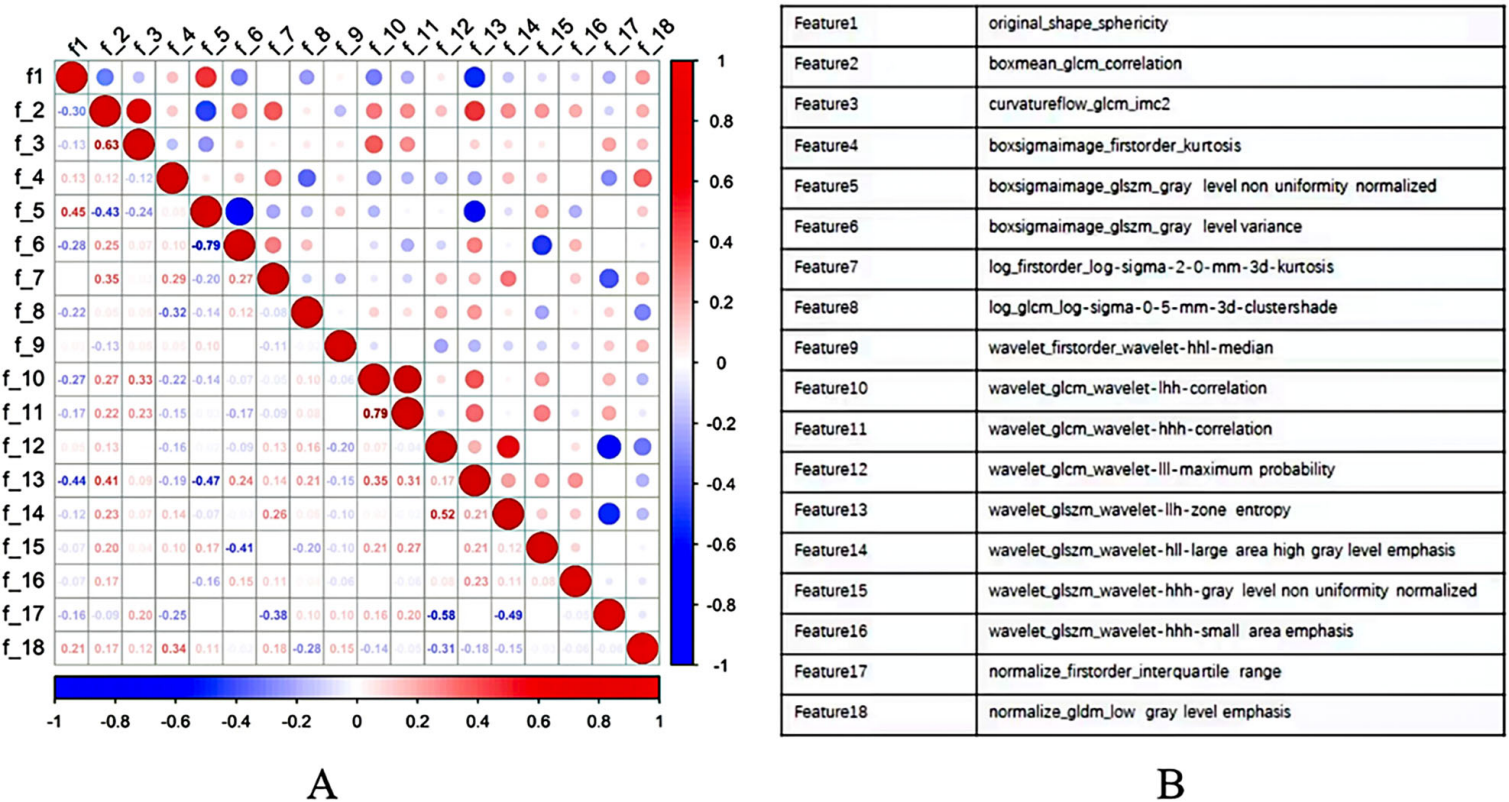
**A** The Pearson correlation map among features; **B** the list of radiomics features

**Fig. 5:**
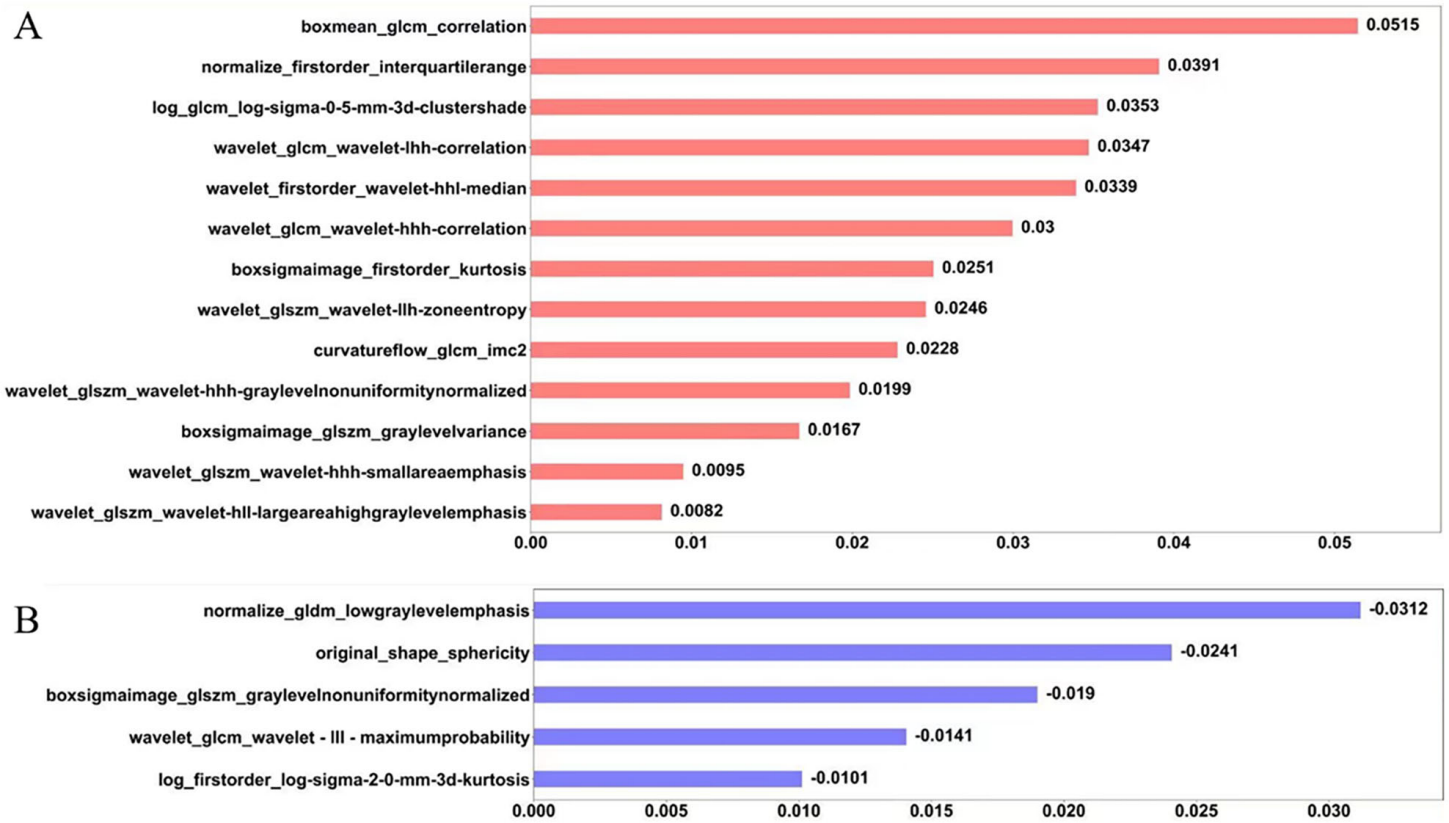
Coefficient of the significant radiomics features. **A**, **B** Represent positive and negative correlated features, respectively

A total of 18 optimal feature subsets were selected, including 1 shape feature, 13 texture features, and 4 first-order statistics features. Pearson correlation analysis between significant radiomics features and the TLS-H group are shown in Fig. 4 and the coefficient of the corresponding features are shown in Fig. 5.

### Performance of radiomics models and clinical models in the discrimination of TLSs

**Fig. 6:**
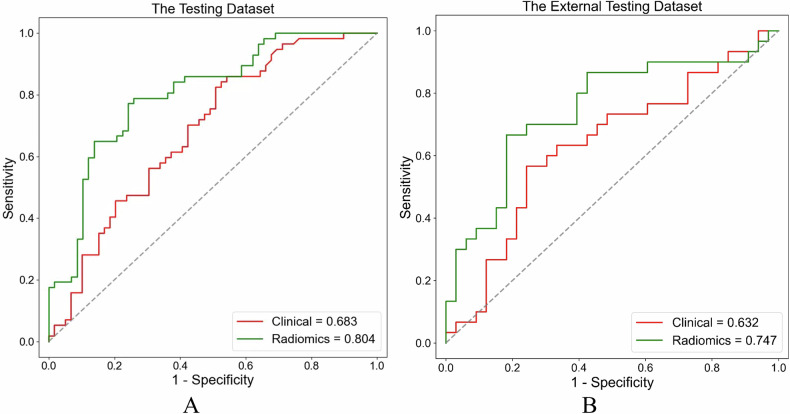
The ROC curves of the internal (**A**) and external (**B**) testing set between radiomics and clinical characteristics

Diagnostic efficiency in radiomics models and clinical models are shown in Table 5. Figure 6 shows the ROC curves of the testing (internal and external) set between radiomics models and clinical models (*p* < 0.001). In the internal testing group, the AUC was 0.804 (95% CI: 0.723–0.884), which was 17.7% higher than the clinical model (AUC: 0.683, 95% CI: 0.586–0.780). In the external set, the AUC was 0.747 (95% CI: 0.621–0.874), which was higher than the clinical model (AUC: 0.632, 95% CI: 0.491–0.774).

**Table 5:** Results of ablation analyses on train, internal, and external test datasets

Parameter	Training data set	Internal test data set		External test data set	
	Radiomics	Radiomics	Clinical	Radiomics	Clinical
AUC (95% CI)	0.781 (0.659–0.905)	0.804 (0.723–0.884)	0.683 (0.586–0.780)	0.747 (0.621–0.874)	0.632 (0.491–0.774)
SEN	0.692	0.789	0.607	0.667	0.633
SPE	0.766	0.707	0.593	0.818	0.576
ACC	0.734	0.748	0.600	0.746	0.603
Precision	0.700	0.726	0.586	0.769	0.576
FI-score	0.689	0.756	0.596	0.714	0.603

*AUC* area under the curve, *CI* confidence interval

## Discussion

This study illuminates the existence of distinct profiles of TLSs in IA, showing their ubiquitous presence in all stages. We built a radiomics model with 18 features based on the SVM algorithm to determine the status of TLSs in patients with IA. Compared to the clinical model, the radiomics model demonstrated the best-discriminating performance in internal and external test cohorts, suggesting that the radiomics signature is a non-invasive biomarker for discriminating TLSs in patients with IA.

In this study, most lesions are located in the upper lobes. In the TLS-H group the upper lobe of the right lung accounts for 35.03%, while in the TLS-L group the upper lobe of the left lung accounts for 35.02%. Our findings are consistent with previous studies that lung cancer occurs more frequently in the upper lobes regardless of the presence of TLS, and the presence of TLS was independent of the type of nodule (ground glass nodule and SN) [21, 22]. Previous studies have suggested that vascular proliferation, fibrosis, and alveolar cavity collapse contribute to the solid components in the lung nodules, which reflect the invasiveness of IA [23]. While Ren et al [24] found that an increase in the solid component of lung nodules was not only associated with an increase in tumor cells, but also with an increase in the number of TLS. However, previous studies have also suggested that TLS plays a positive role in the anti-tumor process, is associated with a positive prognosis in many cancers, and has been shown to be usually independent of the tumor node metastasis stage of the tumor [25], suggesting a two-sided nature of the increase in the solid component of lung tumor.

Among the internal test set, the CT values and maximum nodule diameters in the TLS-H group were significantly higher than those in the TLS-L group. Studies have shown that with increasing numbers and deeper infiltration, tumor cells can replace normal lung tissue to thicken the alveolar space, causing alveolar collapse, reducing gas in the alveolar cavity, and carrying more solid components, which leads to higher density and causing an increased CT value [26]. In this study, the increase in CT value in lung nodules was not only related to the increase in tumor cells, but also to the increase in the number of TLSs, which was consistent with a previous study [24]. In the traditional diagnosis, the CT value and the size of the nodule are often used as the evidence for the diagnosis, and the higher CT value and the larger nodule diameter imply higher aggressiveness and poorer prognosis, but the presence of TLS suggests the importance of evaluating TLS simultaneously. Air bronchograms were more likely to occur in the TLS-H group than in the TLS-L group in the test set. The air bronchogram refers to thin strips of air with a diameter of about 1 mm in the lesion or small alveolar shadows on successive adjacent levels. The pathologic basis is dilated bronchioles and is thought to be closely related to histopathologic infiltration histopathological infiltration. This finding also does not necessitate a poor prognosis when there is a presence of TLSs.

The regression analysis showed that the CT value and the air bronchogram were independent predictors for determining the status of TLSs in the test set. If a nodule has a higher CT value, a larger nodule diameter, or an air bronchogram, we may need to determine whether the prognosis is poor based on TLSs. However, we still cannot say that the prognosis is poor just because TLSs are present. The beneficial effect of TLSs on tumors is positively correlated not only with their density but also with their maturity in the tumor area [6, 27–29]. The higher the number of TLS, the higher the maturity of TLS, and the better the clinical outcomes and responses to immunotherapy [30]. In this study, we consider the number of TLS, TLS maturity should be considered in further study.

Although TLS has been identified in many cancer types and has a well-defined prognostic value, difficulties still remain in the definition and characterization of TLS. There are few studies that have used radiomics to predict the status of TLSs in patients with IA. In this study, the diagnostic efficiency of the SVM algorithm is superior to that of other algorithms. This may be due to its ability to better handle the problem of uneven data distribution when solving small samples, nonlinear, high-dimensional regression, and binary classification problems [31]. The external test set of our model also has better diagnostic performance, indicating that our radiomic model is robust across different machines. This two-center study utilizes the radiomics model to determine the status of TLSs and achieved better performance than the visual assessment by thoracic radiologists. The mean CT value, maximum nodule diameter, and morphological features on CT are the methods most commonly used in routine clinical practice for pulmonary nodule evaluation. However, it may depend on the radiologist’s ability and experience to recognize the signs, which may explain why the AUC for morphological characteristics was lower than that of the radiomics signature in this study.

Our study has several limitations. First, it is a retrospective study that may carry inherent biases. Second, the radiomics features were derived from automatic segmentation, which could not exclude internal vessels and bronchi, potentially affecting the ACC of some features. Finally, future research should include a larger range of cases based on nodule type, TLS maturity, and consolidation radiographic morphology of partially SNs.

In conclusion, the radiomics signature can provide a robust, non-invasive, low-cost, and repeatable method for preoperative differentiation of the status of TLSs in patients with IA, which could help deepen the understanding of image signs, highlight its potential in informing cancer prognosis and guide the selection of clinical treatment in IA.

## Supplementary information

ELECTRONIC SUPPLEMENTARY MATERIAL

## Data Availability

The data presented in this study are available on request from the corresponding author. The data are not publicly available due to hospital regulations.

## Abbreviations

ACC: Accuracy
AUC: Area under curve
CI: Confidence intervals
GGN: Ground-glass nodule
IA: Invasive pulmonary adenocarcinoma
ICC: Intraclass correlation coefficient
OR: Odds ratio
ROC: Receiver operating characteristic
SEN: Sensitivity
SN: Solid nodule
SPE: Specificity
SVM: Support vector machine
TLS: Tertiary lymphoid structures
