# Supplementary material for: Determining the status of tertiary lymphoid structures in invasive pulmonary adenocarcinoma based on chest CT radiomic features
Source: Insights Imaging. 2025 Jan 29;16:28. doi: 10.1186/s13244-025-01906-w (PMC11780022; doi:10.1186/s13244-025-01906-w)
Supplement: Supplementary file 1 — ELECTRONIC SUPPLEMENTARY MATERIAL [file 13244_2025_1906_MOESM1_ESM.pdf]

Determining the Status of Tertiary Lymphoid Structures in Invasive Pulmonary Adenocarcinoma Based on Chest CT Radiomic Features

ELECTRONIC SUPPLEMENTARY MATERIAL

Table S1 Inter-observer agreement of variates.

Variates	ICC (95% CI)
Maximum nodule diameter	0.992（0.986-0.995）
Mean CT value	0.941（0.916-0.959）
Spiculation	0.786（0.703-0.848）
Lobulation	0.816（0.743-0870）
Air bronchogram	0.913（0.876-0.939）
Pleural indentation	0.907（0.869-0.935）
Vascular convergence	0.749（0.652-0.822）
Vacuole sign	0.904（0.865-0.933）
Vascular going through	0.790（0.710-0.849）
Interface	0.793（0.714-0.852）

ICC intraclass correlation coefficient, *CI* confidence interval
